# Mitochondrial Functionality Is Regulated by Alkylphospholipids in Human Colon Cancer Cells

**DOI:** 10.3390/biology12121457

**Published:** 2023-11-22

**Authors:** Margalida Torrens-Mas, Alejandro Collado-Solé, Alberto Sola-Leyva, María Paz Carrasco-Jiménez, Jordi Oliver, Daniel Gabriel Pons, Pilar Roca, Jorge Sastre-Serra

**Affiliations:** 1 Grupo Multidisciplinar de Oncología Traslacional, Research Institute of Health Sciences (IUNICS), University of Balearic Islands, 07122 Palma de Mallorca, Spain; lida.torrens@uib.es (M.T.-M.); jordi.oliver@uib.es (J.O.); d.pons@uib.es (D.G.P.); jorge.sastre@uib.es (J.S.-S.); 2 Departamento de Bioquímica y Biología Molecular I, Facultad de Ciencias, University of Granada, Av. Fuentenueva s/n, 18001 Granada, Spain; albertosola@ugr.es (A.S.-L.); mpazcj@ugr.es (M.P.C.-J.); 3 Health Research Institute of the Balearic Islands (IdISBa), Hospital Universitario Son Espases, Edificio S, 07120 Palma de Mallorca, Spain; 4 Ciber Fisiopatología Obesidad y Nutrición (CB06/03), Instituto Salud Carlos III, 28029 Madrid, Spain

**Keywords:** Alkylphospholipids, perifosine, colorectal cancer, mitochondrial function

## Abstract

**Simple Summary:**

Alkylphospholipids (APLs) are compounds currently under investigation due to their potential to target cancer cells and inhibit their growth. Although their mechanism of action is not fully understood, it is well known that they interfere with the way cancer cells manage membrane lipid metabolism, particularly affecting their phosphatidylcholine and cholesterol levels. Mitochondria, essential cellular organelles, require cholesterol for proper functioning, which involves energy production for the cell. Therefore, our aim was to analyze whether APLs could also interfere with mitochondria in colorectal cancer cells. Our findings indicate that APLs, especially perifosine, alter mitochondrial function and lead to an increase in toxic subproducts that damage cells. Consequently, this treatment reduces the viability of colorectal cancer cells by interfering with mitochondrial function.

**Abstract:**

Alkylphospholipids (APLs) have been studied as anticancer drugs that interfere with biological membranes without targeting DNA. Although their mechanism of action is not fully elucidated yet, it is known that they disrupt the intracellular trafficking of cholesterol and its metabolism. Here, we analyzed whether APLs could also interfere with mitochondrial function. For this purpose, we used HT29 colorectal cancer cells, derived from a primary tumor, and SW620 colorectal cancer cells, derived from a metastasis site. After treatment with the APLs miltefosine and perifosine, we analyzed various mitochondrial parameters, including mitochondrial mass, cardiolipin content, mitochondrial membrane potential, H_2_O_2_ production, the levels of oxidative phosphorylation (OXPHOS) complexes, metabolic enzymes activity, the oxygen consumption rate, and the levels of apoptosis and autophagy markers. APLs, especially perifosine, increased mitochondrial mass while OXPHOS complexes levels were decreased without affecting the total oxygen consumption rate. Additionally, we observed an increase in pyruvate dehydrogenase (PDH) and isocitrate dehydrogenase (IDH) levels and a decrease in lactate dehydrogenase (LDH) activity, suggesting a metabolic rewiring induced by perifosine. These alterations led to higher mitochondrial membrane potential, which was potentiated by decreased uncoupling protein 2 (UCP2) levels and increased reactive oxygen species (ROS) production. Consequently, perifosine induced an imbalance in mitochondrial function, resulting in higher ROS production that ultimately impacted cellular viability.

## 1. Introduction

Alkylphospholipids (APLs) are a class of anticancer drugs that inhibit the proliferation of cancer cells without targeting the DNA [1]. Their chemical structure, similar to lysophosphatidylcholines (lysoPCs), enables them to interfere with cellular membranes [1,2], acting as detergents and inducing apoptosis [3,4]. APLs seem to exhibit higher specificity toward cancer cells, as they show higher uptake of these compounds compared to normal cells and exhibit higher apoptosis rates [1]. They have been tested in a wide variety of cancers, including breast cancer [5,6], colon cancer [7,8,9], hepatoblastoma and glioblastoma [10,11], and hematologic malignancies [12,13], among others. In fact, they have been assessed in combination with chemotherapeutic drugs or radiotherapy, obtaining better outcomes compared to separate treatments [1,8,14,15].

APLs are chemically categorized into two groups: alkyl-lysophospholipids and alkylphosphocholines (APC). The most common APCs are miltefosine (first generation), which is used as a validated drug [16], and perifosine (second generation) [4]. Perifosine (D-21266 octadecyl-(1,1-dimethyl-piperidino-4-yl)-phosphate) is an APC that has undergone structural modification to improve the therapeutic potency of miltefosine (2-(hexadecoxy-oxido-phosphoryl)oxyethyl-trimethyl-azanium) [1,4]. This modification involves a heterocyclic piperidine group replacing the choline unit, increasing the drug’s stability and preventing rapid metabolic degradation by phospholipases [1].

The mechanism behind apoptosis induction by APLs is not yet fully elucidated. It has been reported that APLs alter the cholesterol transport from the plasma membrane to the endoplasmic reticulum, preventing its esterification [1]. Thus, APLs stimulate intracellular cholesterol synthesis and upregulate genes involved in extracellular cholesterol uptake [11]. Via this mechanism, APLs ultimately elevate intracellular cholesterol levels while reducing the synthesis of choline-containing phospholipids, interfering with membrane fluidity [1]. Additionally, APLs also affect membrane-dependent signaling pathways such as PI3K-Akt, Raf-Erk1/2 [3], or phospholipase C [17], possibly due to their accumulation in membranes and the disruption of lipid rafts domains [18,19]. Furthermore, APLs may also impede cell proliferation by blocking autophagy flux, impeding the fusion of autophagosomes with lysosomes [10].

APLs also interfere with the membranes of organelles, including mitochondria [20]. While mitochondria possess a low content of cholesterol in their membranes, this content is tightly regulated [21]. It has been reported that solid tumors increase cholesterol content in mitochondrial membranes, which correlates with tumor malignancy and increased resistance to apoptosis [22]. This elevation in cholesterol can lead to mitochondrial dysfunction, enhancing the Warburg effect in cancer cells. Indeed, perifosine has been described to affect oxidative phosphorylation in isolated mitochondria [2].

The aim of this study was to determine the effect of miltefosine and perifosine on human colon cancer cells, especially analyzing their possible impact on mitochondrial function. HT29, a primary cancer cell line, and SW620, a metastatic cancer cell line, were employed to investigate whether APLs may exert differential effects on cancer cells depending on their cancer metastatic potential. For this purpose, we first assessed the effect of these APLs on cell viability, mitochondrial parameters, and reactive oxygen species (ROS) production, followed by a detailed analysis of the effects of perifosine on mitochondrial metabolism. Our findings indicate that APLs, specifically perifosine, alter mitochondrial mass and function, which may lead to a metabolic switch and increased ROS production, ultimately affecting cell viability.

## 2. Materials and Methods

### 2.1. Reagents

Dulbecco’s Modified Eagle’s Medium (DMEM) high glucose was obtained from GIBCO (Paisley, UK). Fetal bovine serum (FBS) and penicillin/streptomycin were purchased from Biological Industries (Kibbutz Beit Haemek, Israel). Perifosine and miltefosine were obtained from the group of Biomembranes (CTS-236) of the University of Granada. Routine chemicals were supplied by Sigma-Aldrich, Panreac (Barcelona, Spain), Thermo Fisher (Waltham, MA, USA), and Bio-Rad Laboratories (Hercules, CA, USA).

### 2.2. Cell Culture and Treatments

Human colon cancer cell lines HT29 and SW620 were purchased from the American Type Culture Collection (ATCC; Manassas, VA, USA). HT29 cells are derived from a primary colorectal adenocarcinoma, while SW620 are derived from a metastasis in a lymph node. Cells were routinely cultured in DMEM supplemented with 10% FBS and 1% (*v/v*) penicillin/streptomycin in a humidified atmosphere at 37 °C with 5% CO_2_.

Perifosine and miltefosine were dissolved in sterile H_2_O, creating a 5 mM stock solution, which was aliquoted and stored at −20 °C until use and thawed a maximum of two times. For experiments involving fluorimetry assays, cells were seeded in 96-well plates at a density of 5 × 10^4^ cells/well for the HT29 cell line and 4 × 10^4^ cells/well for the S6W20 cell line. For the rest of the experiments, cells were seeded in 6-well plates at a density of 1.5 × 10^6^ cells/well. The next day, cells were treated with the indicated concentrations of vehicle (sterile H_2_O), miltefosine, or perifosine for 48h.

### 2.3. Determination of Cell Viability

After treatment, cell viability was determined via DNA staining with Hoechst 33342. Briefly, cell culture media was removed, and cells were rinsed once with sterile PBS. Then, 0.5 µg/well Hoechst 33342 was incubated for 5 min at 37 °C with 5% CO_2_. Fluorescence was measured in an FLx800 microplate reader (BIO-TEK, Winooski, VT, USA) set at 360/40 nm and 460/40 nm excitation and emission wavelengths, respectively.

### 2.4. Analysis of Mitochondrial Parameters

MitoTracker™ Green (MTG, #M7514, Invitrogen, Waltham, MA, USA) was used to estimate mitochondrial mass, and cardiolipin content was analyzed using the fluorometric probe nonyl acridine orange (NAO, Å1372, Invitrogen). These measurements were conducted as exposed in [23]. Mitochondrial membrane potential was determined with the tetramethylrhodamine methyl ester probe (TMRM, #T668, Invitrogen). Cells were incubated with 10 nM TMRM for 15 min at 37 °C with 5% CO_2_. Then, fresh media was added, and cells were incubated for 10 more min. Finally, cells were rinsed with sterile PBS, and fluorescence was measured in an FLx800 microplate reader (BIO-TEK, Winooski, VT, USA). The excitation wavelength was set at 530/20 nm and the emission wavelength at 590/20 nm. All fluorescence values were normalized per cell viability determined by Hoechst 33342, as described previously.

### 2.5. Measurement of H_2_O_2_ Production

Amplex^®^ Red Hydrogen Peroxide/Peroxidase Assay kit (#A22188, Invitrogen) was used to determine H_2_O_2_ production as described before [24]. Briefly, cells were incubated with 50 µM Amplex Red and 0.1 U/mL HRP in Krebs–Ringer buffer, and fluorescence was recorded at different times. Values were normalized with cell viability determined by Hoechst 33342.

### 2.6. Western Blot

After APL treatment, cells were scraped into 200 µL of RIPA buffer (50 mM Tris-HCl pH 7.5, 150 mM NaCl, 0.1% SDS, 0.5% deoxycholate, and 1% Triton X-100, 10 mM EDTA) and protease and phosphatase inhibitors (10 µM leupeptin, 10 µM pepstatin, 2 mM PMSF, 1 mM NaF, and 1 mM Na_3_VO_4_). Cells were then sonicated on ice at 40% amplitude for 10 s three times (Vibra Cell Ultrasonic Processor 75185). Samples were then centrifuged at 14,000× *g* for 10 min at 4 °C, and protein content was determined with the BCA assay kit (#23227, Pierce, Bonn, Germany) following the manufacturer’s instructions.

For Western Blots, 35 µg of protein were separated into SDS-PAGE gels, preparing 15% resolving gels of 30% acrylamide/bis solution for the OXPHOS complexes and 12% resolving for the other proteins. Then, proteins were electrotransferred onto nitrocellulose membranes with the Trans-blot Turbo system (Bio-Rad) and incubated in a blocking solution of 5% non-fat powdered milk in TBS-Tween (0.05% Tween 20) for 1 h. The following antibodies were incubated overnight at 4 °C: total OXPHOS human WB antibody cocktail (1:500 dilution, #MS601, Abcam, Orlando, FL, USA); PARP (1:1000 dilution, 46D11, #9542, Cell Signaling Technology, Danvers, MA, USA); IDH2 (1:1000 dilution, D7H6Q, #12652, Cell Signaling Technology, Danvers, MA, USA); LC3A/B (1:1000 dilution, #4108, Cell Signaling Technology, Danvers, MA, USA); UCP2 (1:1000 dilution, G-6, #sc-390189, Santa Cruz Biotechnology, Santa Cruz, CA, USA); PDH-E1α (1:500 dilution, D-6, sc-377096, Santa Cruz Biotechnology, Santa Cruz, CA, USA); GAPDH (1:500 dilution, FL-335, sc-25778, Santa Cruz Biotechnology, Santa Cruz, CA, USA). Protein bands were visualized with ImmunStar™ Westen C™ Chemiluminescent Kit Western blotting detection systems using a Chemidox XRS densitometer (Bio-Rad). Results were analyzed using Quantity One Software (version 4.6.6, Bio-Rad). GAPDH was used as a loading control for all blots.

### 2.7. Enzymatic Activities

Cells were harvested by scraping them into 200 µL of STE (Sodium Chloride-Tris-EDTA) buffer as described in [25]; enzymatic activities were analyzed immediately after with different spectrophotometric assays. Cytochrome c oxidase (COX) and lactate dehydrogenase (LDH) activities were determined as described before [25].

### 2.8. Oxygen Consumption Rate (OCR)

The real-time oxygen consumption rate was determined using the Seahorse Extracellular Flux (XFe96) analyzer (Seahorse Bioscience, Billerica, MA, USA) as described in [23]. Cells were seeded at a density of 1.8 × 10^4^ cells/well in XFe96 plates and incubated overnight at 37 °C with 5% CO_2_. The next day, cells were treated with perifosine for 48 h. For OCR determination, the following inhibitors were used: 1 µM oligomycin, 2 µM FCCP, 0.5 µM rotenone, and 0.5 µM antimycin A. To normalize OCR values by cell viability, a parallel plate was seeded, and cell viability was determined with Hoechst 33342, as described before.

### 2.9. Statistical Analyses

The Statistical Program for the Social Sciences software (SPPS, version 24.0; Chicago, IL, USA) was used for all statistical analyses. Results are presented as means ± standard error of the mean (SEM). Statistical differences between control and miltefosine-treated cells and control and perifosine-treated cells were analyzed using Student’s *t*-test. Statistical significance was set at *p*-value < 0.05.

## 3. Results

### 3.1. APLs Decrease Cell Viability in Colon Cancer Cells

**Figure 1:**
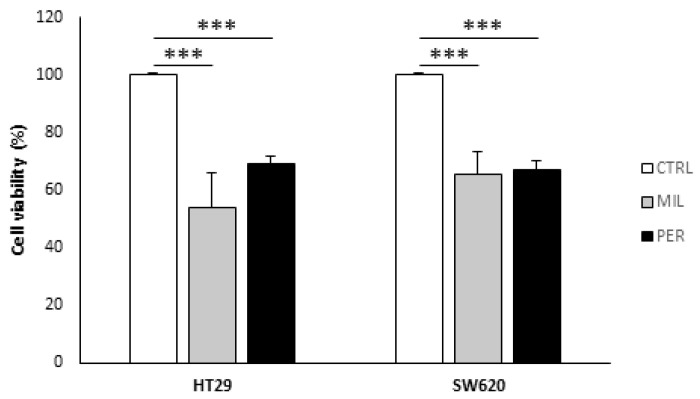
Cell viability was reduced after APL treatment. Cell viability was analyzed via Hoechst 33342 staining. Cells were treated with vehicle (“CTRL”, white bars), 100 µM miltefosine (“MIL”, grey bars), or 60 µM perifosine (“PER”, black bars). Results are presented as the mean ± SEM (*n* = 6), and control cells were set at 100%. Student’s *t*-test was performed to check statistical differences: *** *p*-value < 0.001.

We first assessed cell viability in HT29, a primary colon cancer cell line, and SW620, a metastatic colon cancer cell line. We observed a reduction in cell viability of 30–40% in both cell lines at 100 µM miltefosine and 60 µM perifosine (Figure 1).

### 3.2. APLs Increase Mitochondrial Mass and H_2_O_2_ Production

**Figure 2:**
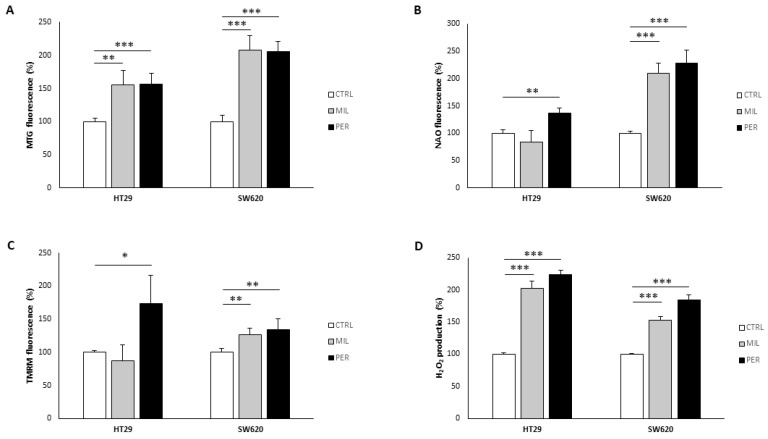
APL treatment increases mitochondrial mass and reactive oxygen species production. (**A**) Mitotracker™ Green (MTG) fluorescence; (**B**) Nonyl acridine orange (NAO) fluorescence; (**C**) Tetramethylrhodamine methyl ester (TMRM) fluorescence; (**D**) H_2_O_2_ production measured by Amplex Red fluorescence. Cells were treated with vehicle (“CTRL”, white bars), 100 µM miltefosine (“MIL”, grey bars), or 60 µM perifosine (“PER”, black bars). Results are presented as the mean ± SEM (*n* = 6), and control cells were set at 100%. Student’s *t*-test was performed to check statistical differences: * *p*-value < 0.05, ** *p*-value < 0.01, *** *p*-value < 0.001.

Next, we analyzed several mitochondria-related parameters to evaluate mitochondrial mass and functionality. Miltefosine and perifosine significantly increased MTG fluorescence (Figure 2A), a marker of mitochondrial mass, to a similar extent in both cell lines. Interestingly, SW620 exhibited higher MTG fluorescence levels after treatment than HT29 (+108.0–105.5% in SW620 and +55.3–57.1% in HT29). On the other hand, NAO fluorescence, a marker of cardiolipin content, was increased after treatment with perifosine in the HT29 line (+36.2%), while it remained unchanged with miltefosine treatment (Figure 2B). In contrast, both treatments significantly increased NAO fluorescence in the metastatic cell line SW620 (+109.6% and +128.5%, respectively).

Then, we evaluated mitochondrial membrane potential with the TMRM probe. (Figure 2C). In HT29 cells, we observed a significant increase in TMRM fluorescence only with perifosine treatment (+73.6%), while SW620 cell showed mildly but significantly higher TMRM fluorescence with both treatments (+26% with miltefosine and +34% with perifosine). Finally, since mitochondria are the main source of ROS, we analyzed H_2_O_2_ production. As expected, H_2_O_2_ production increased in both cell lines after treatments, reaching higher levels with perifosine treatment (+124.2% in HT29 cells and +89.8% in SW620 cells) (Figure 2D). Together, these results suggest that APLs increase mitochondrial mass and functionality, therefore increasing ROS production.

### 3.3. Oxidative Phosphorylation Complexes Are Affected by APL Treatment

**Figure 3:**
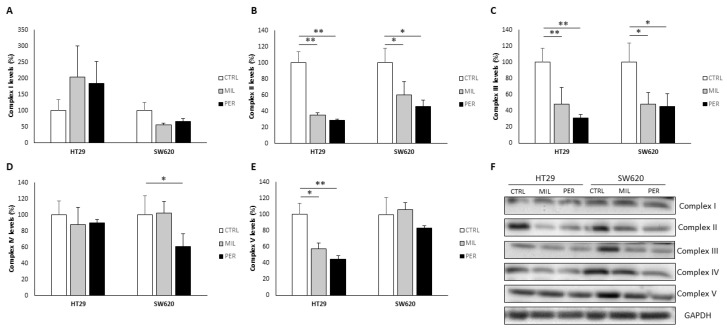
APL treatment changed OXPHOS levels. (**A**) Complex I levels (NDUFB); (**B**) Complex II levels (SDHB); (**C**) Complex III levels (UQCRC2); (**D**) Complex IV levels (COX II); (**E**) Complex V levels (ATP5A); (**F**) Representative bands of all complexes obtained by Western Blot (Original file see Supplementary Materials). GAPDH was used as a loading control. Cells were treated with vehicle (“CTRL”, white bars), 100 µM miltefosine (“MIL”, grey bars), or 60 µM perifosine (“PER”, black bars). Results are presented as the mean ± SEM (*n* = 6), and control cells were set at 100%. Student’s *t*-test was performed to check statistical differences: * *p*-value < 0.05, ** *p*-value < 0.01.

To further check the effect of APLs on mitochondria, we determined the levels of the OXPHOS complexes via Western Blot (Figure 3). Levels of complex I (NADH dehydrogenase, subunit NDUFB8) remained unaffected by treatment; only SW620 cells treated with miltefosine showed a tendency to decrease complex I levels (*p*-value = 0.075). Complexes II (succinate dehydrogenase, subunit 30 kDa) and III (ubiquinol–cytochrome c reductase, subunit Core 2) presented decreased levels in both cell lines with APL treatment, resulting in a reduction of over 40%. In SW620 cells, only complex IV (cytochrome c oxidase, subunit II) was affected by perifosine treatment, showing a 39% reduction in its levels. On the other hand, in HT29 cells, both miltefosine and perifosine treatments resulted in a decrease (−42% and −55%, respectively) in the levels of complex V (ATP synthase, subunit alpha).

Interestingly, when analyzing the complex IV/complex V ratio, both cell lines were affected by APL treatment in a different manner (Table 1). HT29 cells exhibited a higher complex IV/complex V ratio, contrary to SW620 cells, which showed a lower ratio.

### 3.4. Perifosine Alters Mitochondrial Function

Considering that the results obtained so far show similar effects of both APLs on the two different cell lines, and perifosine is a modification of miltefosine that enhances its activity, from now on, we will focus solely on functional analyses using perifosine.

**Figure 4:**
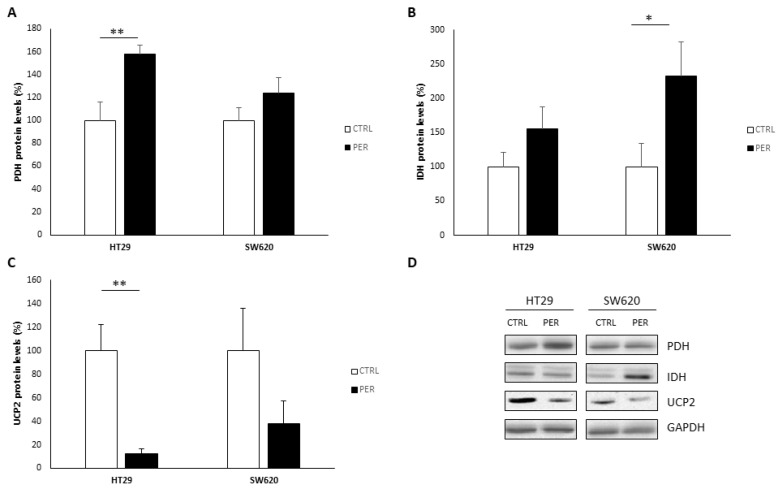
Perifosine affects mitochondrial function at protein level. (**A**) Pyruvate dehydrogenase (PDH) protein levels; (**B**) Isocitrate dehydrogenase (IDH) protein levels; (**C**) Uncoupling protein 2 (UCP2) protein levels; (**D**) Representative bands obtained via Western Blot (Original file see Supplementary Materials). GAPDH was used as a loading control. Cells were treated with vehicle (“CTRL”, white bars) or 60 µM perifosine (“PER”, black bars). Results are presented as the mean ± SEM (*n* = 6), and control cells were set at 100%. Student’s *t*-test was performed to check statistical differences: ** *p*-value < 0.01, * *p*-value < 0.05.

To further check the mitochondrial function, we analyzed the levels of pyruvate dehydrogenase (PDH), isocitrate dehydrogenase (IDH), and uncoupling protein 2 (UCP2). PDH levels were significantly increased in the HT29 cell line in response to perifosine (Figure 4A), while the SW620 cell line increased IDH levels (Figure 4B). Additionally, perifosine significantly reduced the levels of UCP2 in HT29 (Figure 4C), reaching 23% of vehicle-treated cells. We also observed a reduction in UCP2 levels in SW620, although it did not reach statistical significance (*p*-value = 0.055).

Next, we assessed oxygen consumption (Table 2). We did not find any statistically significant changes in basal oxygen consumption, maximal respiration, or proton leak. However, SW620 cells exhibited a dramatic decrease in mitochondrial ATP production.

**Figure 5:**
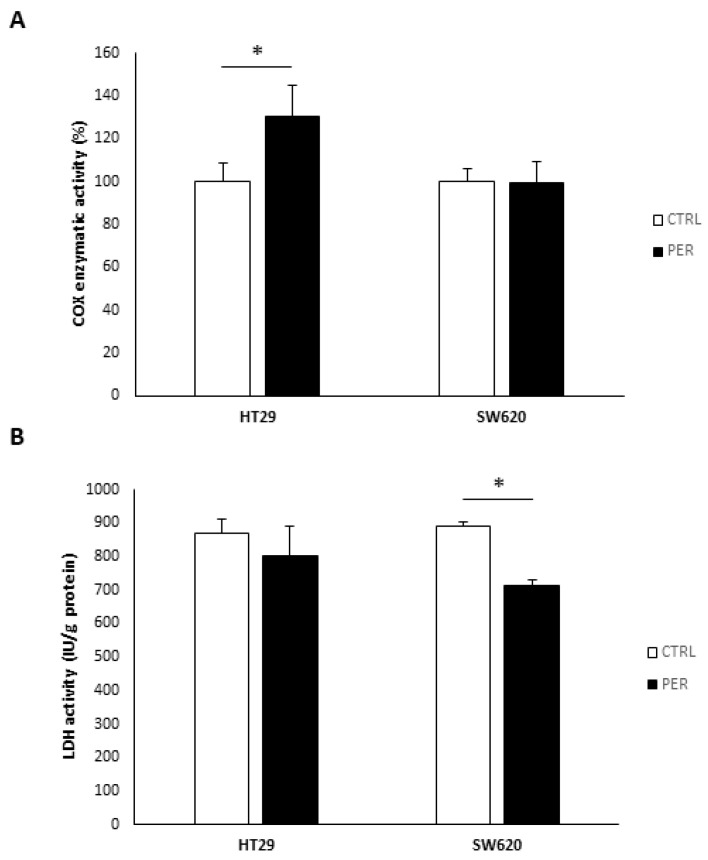
Perifosine alters the enzymatic activities of metabolic enzymes. (**A**) Complex IV (COX) enzymatic activity; (**B**) Lactate dehydrogenase (LDH) enzymatic activity. Cells were treated with vehicle (“CTRL”, white bars) or 60 µM perifosine (“PER”, black bars). Results are presented as the mean ± SEM (*n* = 3) for COX activity, and control cells were set at 100%. For the LDH activity, results are shown as international units (IU) per gram of protein (*n* = 3). Student’s *t*-test was performed to check statistical differences: * *p*-value < 0.05.

Lastly, Figure 5 shows the enzymatic activities of complex IV (COX) and lactate dehydrogenase (LDH). We found that in HT29 cells, COX activity was increased by 30.8% after perifosine treatment, while in SW620, despite the decrease in protein levels, COX enzymatic activity was preserved. Moreover, SW620 cells also showed decreased LDH enzymatic activity after treatment.

Altogether, these results suggest that, despite the decrease in complex V levels in HT29 cells after perifosine treatment, ATP production is preserved, presumably due to the reduced levels of UCP2. This reduction in UCP2 may lead to a better coupling efficiency of the respiratory chain, enhancing ATP synthase activity. On the other hand, in SW620 cells, perifosine reduced the ratio complex IV/complex V, and although UCP2 levels also seemed to be decreased, mitochondrial ATP production dropped drastically.

### 3.5. Perifosine Increases PARP Cleavage and LC3-II Levels

**Figure 6:**
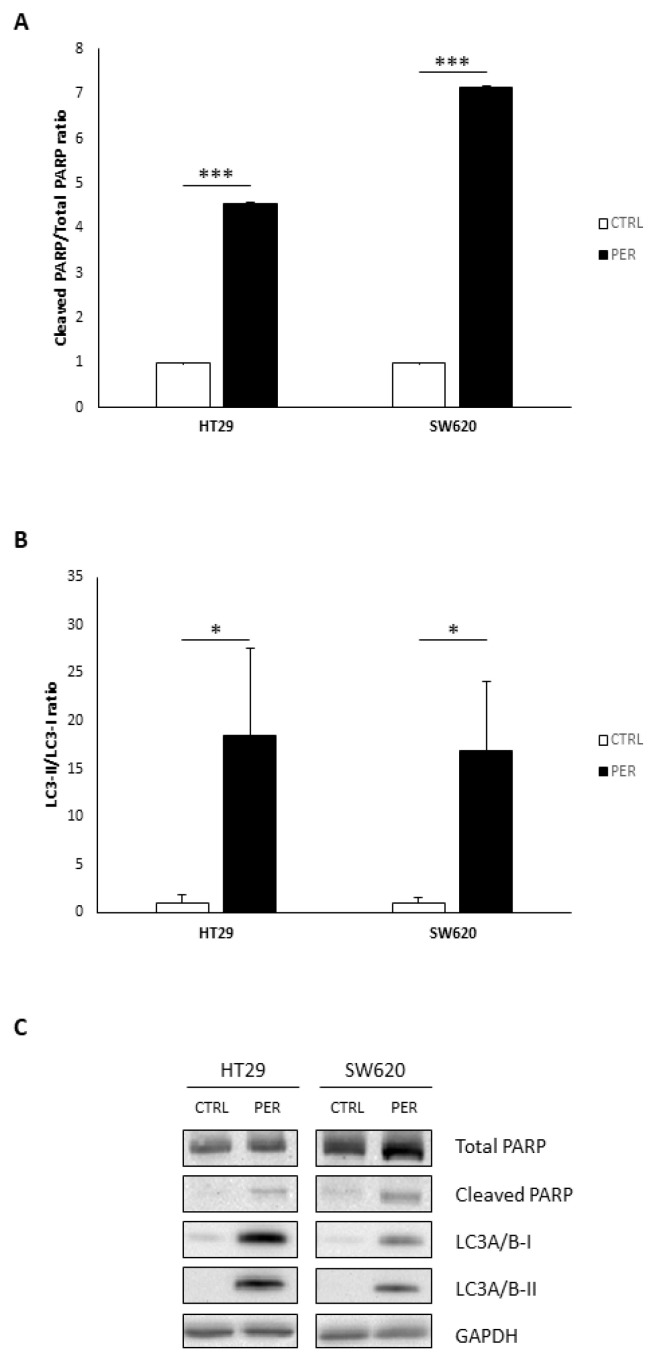
Perifosine increases markers of apoptosis and autophagy. (**A**) Cleaved PARP/Total PARP ratio; (**B**) LC3A/B-II/LC3A/B-I ratio; (**C**) representative bands obtained via Western Blot (Original file see Supplementary Materials). GAPDH was used as a loading control. Cells were treated with vehicle (“CTRL”, white bars) or 60 µM perifosine (“PER”, black bars). Results are presented as the mean ± SEM (*n* = 6), and control cells were set at 100%. Student’s *t*-test was performed to check statistical differences: * *p*-value < 0.05; *** *p*-value < 0.001.

Finally, we analyzed PARP cleavage as a marker of apoptosis and LC3-II as a marker of autophagy to further assess the effects of perifosine (Figure 6). Figure 6A,C show a great increase in the levels of cleaved PARP, resulting in a higher cleaved PARP/total PARP ratio after treatment with perifosine. On the other hand, the LC3-II/LC3-I ratio also increased in both cell lines after perifosine treatment (Figure 6B,C), also suggesting an increase in autophagy.

## 4. Discussion

**Figure 7:**
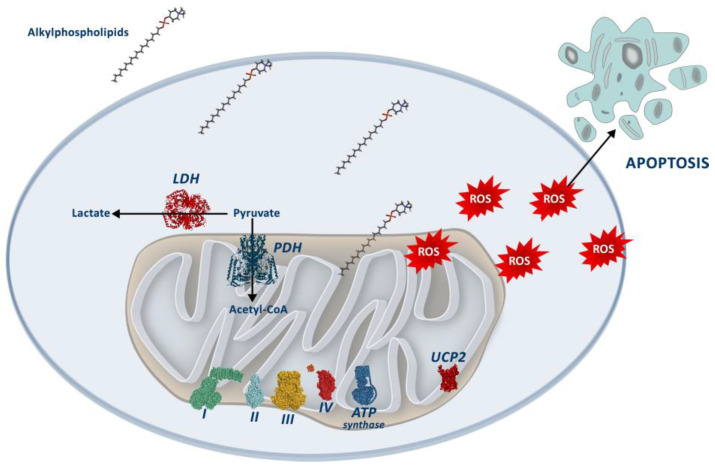
Summary of the effects of Alkylphospholipids, with a special focus on perifosine on colon cancer cells. APLs interfere with mitochondrial function, presumably inducing a metabolic change and leading to an increase in ROS production, which decreases cell viability via an increase in apoptosis.

In this study, we showed that APLs in colorectal cancer cell lines decrease cell viability and alter the mitochondrial pool, increasing mitochondrial mass, cardiolipin content, and mitochondrial membrane potential, ultimately increasing ROS production. Concretely, perifosine treatment produced a general decrease in the protein levels of the OXPHOS complexes, although the basal oxygen consumption rate was unaltered. Furthermore, perifosine increased the levels of PDH and IDH while decreasing the levels of UCP2 and the activity of COX and LDH, suggesting a metabolic rewiring. These results are summarized in Figure 7.

We have shown here that APLs produce significant changes in the mitochondrial pool. Concretely, APL-treated cells showed an increase in mitochondrial mass, as seen by the rise in MTG fluorescence. Indeed, the cardiolipin content, which is the major phospholipid of the mitochondrial inner membrane [26], was also higher after APL treatment. Cardiolipin enhances the activity of some complexes of the mitochondrial respiratory chain, especially complex IV and III, and has been described to facilitate the assembly and stabilization of supercomplexes [26,27]. Despite the general decrease in the protein levels of OXPHOS, we did not observe any major changes in mitochondrial oxygen consumption, suggesting a preservation of their enzymatic activity. Thus, this increase in cardiolipin may be a compensatory mechanism to overcome the reduced levels of OXPHOS complexes and maintain the respiratory chain function via supercomplex formation and stability.

On the other hand, it is known that interfering with the quantity of cholesterol in mitochondrial membranes could lead to alterations in mitochondrial function [22,28]. As APLs and, concretely, perifosine have shown an interference with intracellular cholesterol trafficking [1], mitochondrial cholesterol could be affected by this treatment. In fact, treatment with perifosine has previously shown mitochondria with more dilated cristae [10], suggesting mitochondrial function alterations.

In this regard, we have observed an increase in the mitochondrial membrane potential in both cell lines, along with a severe downregulation of UCP2 levels. The membrane potential is generated by the OXPHOS complexes, specifically by the proton-pumping mechanism of complexes I, III, and IV. Then, the complex V (ATP synthase) uses the electrochemical gradient generated to synthesize ATP from ADP and phosphate [29]. On the other hand, one of the functions of UCP2 is to facilitate the reentry of protons into the mitochondrial matrix, bypassing the complex V [30]. In fact, UCP2 has been connected to metabolic rewiring, as it is upregulated in human colon cancer in response to oxidative stress and could be associated with tumor progression [31], so it is expected that cancer cells rely on this protein to maintain their aggressiveness. The preserved oxygen consumption and increase in COX activity could explain the increase in mitochondrial membrane potential observed. Additionally, the dramatic decrease in UCP2 levels we observed after perifosine treatment suggests that the protons pumped by the OXPHOS complexes are retained in the intermembrane space without any alternative pathway for reentering the matrix.

The increase in mitochondrial membrane potential is also correlated with hydrogen peroxide production. In fact, a rise in mitochondrial membrane potential can result in an increase in superoxide anion [29]. Superoxide is then rapidly transformed to H_2_O_2_, considered a biomarker of ROS production in mitochondria, as almost 90% of ROS are produced in these organelles due to the respiratory chain [32]. It has been described that the primary site of generation of ROS are complexes III and I [33]. ROS production can occur when there are highly reduced coenzyme Q levels with maximal mitochondrial membrane potential. In this situation, the reduced coenzyme Q is used by the complex I for the reduction in NAD+ to NADH. This process is known as reverse electron transfer or RET. The reduced coenzyme Q is also oxidized by complex III. Regarding this matter, the treatment with APLs decreased the levels of complex III, which could induce the accumulation of reduced coenzyme Q and the production of ROS involving RET [34]. The level of complex I showed a tendency to increase in HT29 cells after treatment, which could potentiate the effect of RET. In addition, the increase in the mitochondrial membrane potential observed could affect the RET and increase ROS production by the saturation of ATP synthase.

Furthermore, perifosine treatment increased both PDH and IDH levels. PDH is responsible for the entry of pyruvate into the Krebs cycle, while IDH converts isocitrate to alpha-ketoglutarate [35]. The higher levels of these mitochondrial enzymes could increase the Krebs cycle flux, which could be related to the increase observed in other mitochondrial parameters. We also observed a decrease in LDH activity, which could imply an increased pool of acetyl-CoA in the cell, which would presumably be derived from cholesterol synthesis, as has been described before [11].

On the other hand, this study showed some differences in mitochondrial parameters between cell lines. Cardiolipin content and mitochondrial mass were higher in the metastatic cell line SW620 than in the primary tumor cell line HT29 after APL treatment, while mitochondrial ATP production in SW620 cells dropped dramatically. It has been observed that the drug membrane uptake and the effect of ALPs are higher in the malignant state of the cells [8]. Then, it is possible that membrane uptake of perifosine is higher in SW620, explaining the difference in the effects observed. Furthermore, it is known that lipid rafts are distinct between primary and metastatic tumors [36]. As such, APLs can interfere with lipid rafts [19], which could also explain the differential response of the two cell lines.

All these alterations could be responsible, at least in part, for the reduced viability observed after treatment in both cell lines. We have observed an important increase in cleaved PARP and the ratio of LC3-II/I, suggesting an increase in apoptosis and autophagy. Increased content of mitochondrial cholesterol has been linked to apoptosis resistance [22] and the acquisition of chemotherapy resistance [21]. Thus, our results suggest that perifosine could be interfering with mitochondrial function, which could correlate with changes in cholesterol content, leading to higher apoptosis rates. On the other hand, autophagy could be induced by oxidative stress [4], and it is known that perifosine produces a blockage in the autophagy flux that leads to decreased cell proliferation [10]. Thus, our results suggest that perifosine is also interfering with the autophagy process and could be responsible for the decreased cell viability.

## 5. Conclusions

Summing up, mitochondrial functionality is modified by perifosine, resulting in an increase in ROS production that could be related to the imbalance in the OXPHOS complexes. Ultimately, this treatment produces a decrease in cell viability, increasing apoptosis and autophagy.

## Figures and Tables

**Table 1:** Complex IV/Complex V ratio for both cell lines after APL treatment. Results are presented as the mean ± SEM (*n* = 6), and control cells were set at 1. Student’s *t*-test was performed to check statistical differences: * *p*-value < 0.05.

	HT29	SW620
CTRL	1.00 ± 0.08	1.00 ± 0.13
MIL	1.18 ± 0.34	0.81 ± 0.18
PER	1.72 ± 0.37 *	0.66 ± 0.06 *

**Table 2:** Oxygen consumption rate (OCR) after APL treatment. Results are presented as the mean ± SEM in pmol/min (*n* = 3). Student’s *t*-test was performed to check statistical differences: * *p*-value < 0.05.

	HT29		SW620	
	CTRL	PER	CTRL	PER
Basal OCR	46.2 ± 10.2	46.0 ± 16.1	24.6 ± 1.3	15.18 ± 7.9
Maximal respiration	81.2 ± 19.2	53.9 ± 11.0	37.4 ± 0.9	25.7 ± 8.2
Proton leak	46.1 ± 10.8	42.7 ± 16.4	17.4 ± 1.6	17.89 ± 8.8
ATP production	2.05 ± 0.71	3.31 ± 1.39	7.17 ± 0.34	0.77 ± 0.11 *

## Data Availability

Data supporting the findings of this study are available upon request by contacting the corresponding author. This includes raw data for all experiments.

## Supplementary Materials

The following supporting information can be downloaded at: https://www.mdpi.com/article/10.3390/biology12121457/s1, Original uncropped blots.

## References

[1] Ríos-Marco P., Marco C., Gálvez X., Jiménez-López J.M., Carrasco M.P. (2017). Alkylphospholipids: An Update on Molecular Mechanisms and Clinical Relevance. Biochim. Biophys. Acta Biomembr..

[2] Burgeiro A., Pereira C.V., Carvalho F.S., Pereira G.C., Mollinedo F., Oliveira P.J. (2013). Edelfosine and Perifosine Disrupt Hepatic Mitochondrial Oxidative Phosphorylation and Induce the Permeability Transition. Mitochondrion.

[3] Kostadinova A., Topouzova-Hristova T., Momchilova A., Tzoneva R., Berger M.R. (2015). Antitumor Lipids—Structure, Functions, and Medical Applications. Advances in Protein Chemistry and Structural Biology.

[4] Kaleağasıoğlu F., Ali D.M., Berger M.R. (2020). Multiple Facets of Autophagy and the Emerging Role of Alkylphosphocholines as Autophagy Modulators. Front. Pharmacol..

[5] Leighl N.B., Dent S., Clemons M., Vandenberg T.A., Tozer R., Warr D.G., Crump R.M., Hedley D., Pond G.R., Dancey J.E. (2008). A Phase 2 Study of Perifosine in Advanced or Metastatic Breast Cancer. Breast Cancer Res. Treat..

[6] El-Sheridy N.A., El-Moslemany R.M., Ramadan A.A., Helmy M.W., El-Khordagui L.K. (2021). Enhancing the in Vitro and in Vivo Activity of Itraconazole against Breast Cancer Using Miltefosine-Modified Lipid Nanocapsules. Drug Deliv..

[7] Park S., Kim J., Choi J., Lee C., Lee W., Park S., Park Z., Baek J., Nam J. (2021). Lipid Raft-disrupting Miltefosine Preferentially Induces the Death of Colorectal Cancer Stem-like Cells. Clin. Transl. Med..

[8] Morii Y., Tsubaki M., Takeda T., Otubo R., Seki S., Yamatomo Y., Imano M., Satou T., Shimomura K., Nishida S. (2021). Perifosine Enhances the Potential Antitumor Effect of 5-Fluorourasil and Oxaliplatin in Colon Cancer Cells Harboring the PIK3CA Mutation. Eur. J. Pharmacol..

[9] Pavlatovská B., Machálková M., Brisudová P., Pruška A., Štěpka K., Michálek J., Nečasová T., Beneš P., Šmarda J., Preisler J. (2020). Lactic Acidosis Interferes With Toxicity of Perifosine to Colorectal Cancer Spheroids: Multimodal Imaging Analysis. Front. Oncol..

[10] Ríos-Marco P., Ríos A., Jiménez-López J.M., Carrasco M.P., Marco C. (2015). Cholesterol Homeostasis and Autophagic Flux in Perifosine-Treated Human Hepatoblastoma HepG2 and Glioblastoma U-87 MG Cell Lines. Biochem. Pharmacol..

[11] Ríos-Marco P., Martín-Fernández M., Soria-Bretones I., Ríos A., Carrasco M.P., Marco C. (2013). Alkylphospholipids Deregulate Cholesterol Metabolism and Induce Cell-Cycle Arrest and Autophagy in U-87 MG Glioblastoma Cells. Biochim. Biophys. Acta Mol. Cell Biol. Lipids.

[12] Richardson P.G., Nagler A., Ben-Yehuda D., Badros A., Hari P.N., Hajek R., Spicka I., Kaya H., LeBlanc R., Yoon S. (2020). Randomized, Placebo-controlled, Phase 3 Study of Perifosine Combined with Bortezomib and Dexamethasone in Patients with Relapsed, Refractory Multiple Myeloma Previously Treated with Bortezomib. EJHaem.

[13] Jesus L.d.O.P., de Souza A.A., Torquato H.F.V., Gontijo V.S., Pereira de Freitas R., Gesteira T.F., Coulson-Thomas V.J., Torquato R.J.S., Tanaka A.S., Paredes-Gamero E.J. (2022). In Vitro Study of Cytotoxic Mechanisms of Alkylphospholipids and Alkyltriazoles in Acute Lymphoblastic Leukemia Models. Molecules.

[14] Song Z., Tu X., Zhou Q., Huang J., Chen Y., Liu J., Lee S.B., Kim W., Nowsheen S., Luo K. (2019). A Novel UCHL3 Inhibitor, Perifosine, Enhances PARP Inhibitor Cytotoxicity through Inhibition of Homologous Recombination-Mediated DNA Double Strand Break Repair. Cell Death Dis..

[15] Adamová B., Říhová K., Pokludová J., Beneš P., Šmarda J., Navrátilová J. (2023). Synergistic Cytotoxicity of Perifosine and ABT-737 to Colon Cancer Cells. J. Cell Mol. Med..

[16] Villa-Pulgarín J.A., Gajate C., Botet J., Jimenez A., Justies N., Varela-M R.E., Cuesta-Marbán Á., Müller I., Modolell M., Revuelta J.L. (2017). Mitochondria and Lipid Raft-Located FOF1-ATP Synthase as Major Therapeutic Targets in the Antileishmanial and Anticancer Activities of Ether Lipid Edelfosine. PLoS Negl. Trop. Dis..

[17] Marco C., Ríos-Marco P., Jiménez-López J., Segovia J., Carrasco M. (2014). Antitumoral Alkylphospholipids Alter Cell Lipid Metabolism. Anti-Cancer Agents Med. Chem..

[18] Gajate C., Mollinedo F. (2001). The Antitumor Ether Lipid ET-18-OCH3 Induces Apoptosis through Translocation and Capping of Fas/CD95 into Membrane Rafts in Human Leukemic Cells. Blood.

[19] Gomide A.B., Thomé C.H., dos Santos G.A., Ferreira G.A., Faça V.M., Rego E.M., Greene L.J., Stabeli R.G., Ciancaglini P., Itri R. (2013). Disrupting Membrane Raft Domains by Alkylphospholipids. Biochim. Biophys. Acta (BBA)-Biomembr..

[20] Kuerschner L., Richter D., Hannibal-Bach H.K., Gaebler A., Shevchenko A., Ejsing C.S., Thiele C. (2012). Exogenous Ether Lipids Predominantly Target Mitochondria. PLoS ONE.

[21] García-Ruiz C., Ribas V., Baulies A., Fernández-Checa J.C. (2017). Mitochondrial Cholesterol and the Paradox in Cell Death. Handbook of Experimental Pharmacology.

[22] Ribas V., García-Ruiz C., Fernández-Checa J.C. (2016). Mitochondria, Cholesterol and Cancer Cell Metabolism. Clin. Transl. Med..

[23] Martinez-Bernabe T., Sastre-Serra J., Ciobu N., Oliver J., Pons D.G., Roca P. (2021). Estrogen Receptor Beta (ERβ) Maintains Mitochondrial Network Regulating Invasiveness in an Obesity-Related Inflammation Condition in Breast Cancer. Antioxidants.

[24] Alorda-Clara M., Torrens-Mas M., Morla-Barcelo P.M., Roca P., Sastre-Serra J., Pons D.G., Oliver J. (2022). High Concentrations of Genistein Decrease Cell Viability Depending on Oxidative Stress and Inflammation in Colon Cancer Cell Lines. Int. J. Mol. Sci..

[25] Torrens-Mas M., González-Hedström D., Abrisqueta M., Roca P., Oliver J., Sastre-Serra J. (2017). PGC-1α in Melanoma: A Key Factor for Antioxidant Response and Mitochondrial Function. J. Cell Biochem..

[26] Böttinger L., Ellenrieder L., Becker T. (2016). How Lipids Modulate Mitochondrial Protein Import. J. Bioenerg. Biomembr..

[27] Mårtensson C.U., Doan K.N., Becker T. (2017). Effects of Lipids on Mitochondrial Functions. Biochim. Biophys. Acta (BBA)-Mol. Cell Biol. Lipids.

[28] Martin L.A., Kennedy B.E., Karten B. (2016). Mitochondrial Cholesterol: Mechanisms of Import and Effects on Mitochondrial Function. J. Bioenerg. Biomembr..

[29] Zorova L.D., Popkov V.A., Plotnikov E.Y., Silachev D.N., Pevzner I.B., Jankauskas S.S., Babenko V.A., Zorov S.D., Balakireva A.V., Juhaszova M. (2018). Mitochondrial Membrane Potential. Anal. Biochem..

[30] Robbins D., Zhao Y. (2011). New Aspects of Mitochondrial Uncoupling Proteins (UCPs) and Their Roles in Tumorigenesis. Int. J. Mol. Sci..

[31] Kuai X.-Y., Ji Z.-Y., Zhang H.-J. (2010). Mitochondrial Uncoupling Protein 2 Expression in Colon Cancer and Its Clinical Significance. World J. Gastroenterol..

[32] Azadmanesh J., Borgstahl G. (2018). A Review of the Catalytic Mechanism of Human Manganese Superoxide Dismutase. Antioxidants.

[33] Rigoulet M., Yoboue E.D., Devin A. (2011). Mitochondrial ROS Generation and Its Regulation: Mechanisms Involved in H_2_O_2_ Signaling. Antioxid. Redox Signal.

[34] Onukwufor J.O., Berry B.J., Wojtovich A.P. (2019). Physiologic Implications of Reactive Oxygen Species Production by Mitochondrial Complex I Reverse Electron Transport. Antioxidants.

[35] Pavlova N.N., Zhu J., Thompson C.B. (2022). The Hallmarks of Cancer Metabolism: Still Emerging. Cell Metab..

[36] Greenlee J.D., Subramanian T., Liu K., King M.R. (2021). Rafting Down the Metastatic Cascade: The Role of Lipid Rafts in Cancer Metastasis, Cell Death, and Clinical Outcomes. Cancer Res..

