# Supplementary material for: Mitochondrial Functionality Is Regulated by Alkylphospholipids in Human Colon Cancer Cells
Source: Biology (Basel). 2023 Nov 22;12(12):1457. doi: 10.3390/biology12121457 (PMC10740929; doi:10.3390/biology12121457)
Supplement: Supplementary file 1 [file biology-12-01457-s001.zip › biology-2707833-supplementary.pdf]

Blot - OXPHOS complexes (Figure 3)

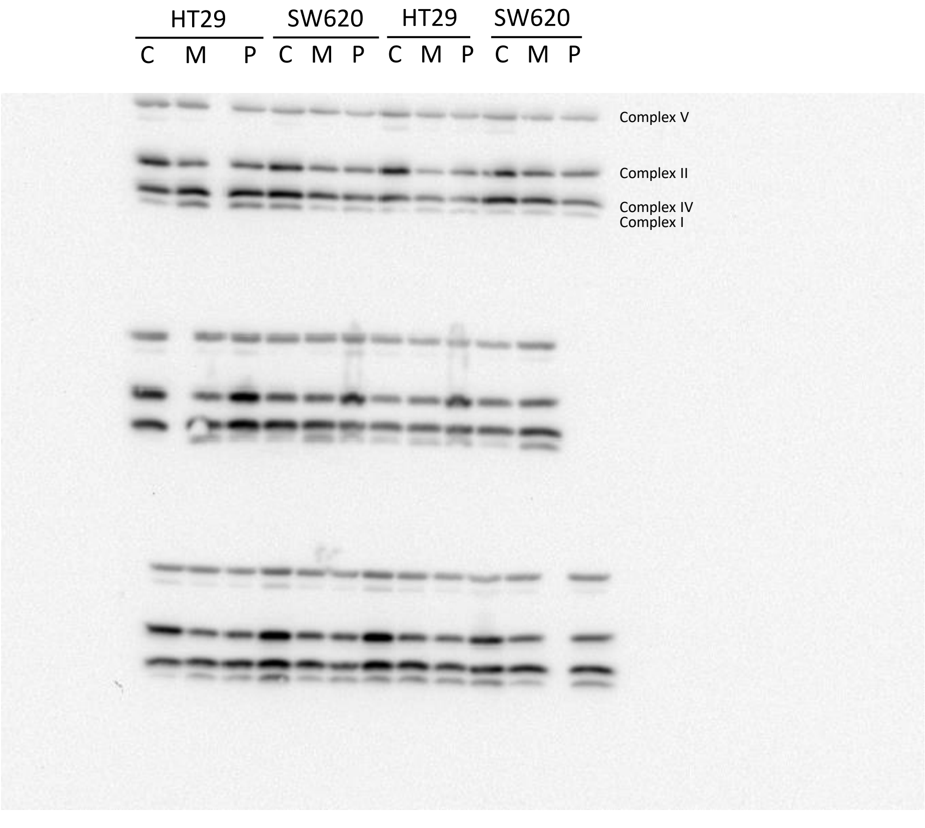

C: vehicle-treated cells
M: miltefosine-treated cells
P: perifosine-treated cells

3 gels blotted into the same membrane. All gels follow the same order.

Blot - OXPHOS complexes (Figure 3)
Further exposition for Complex III

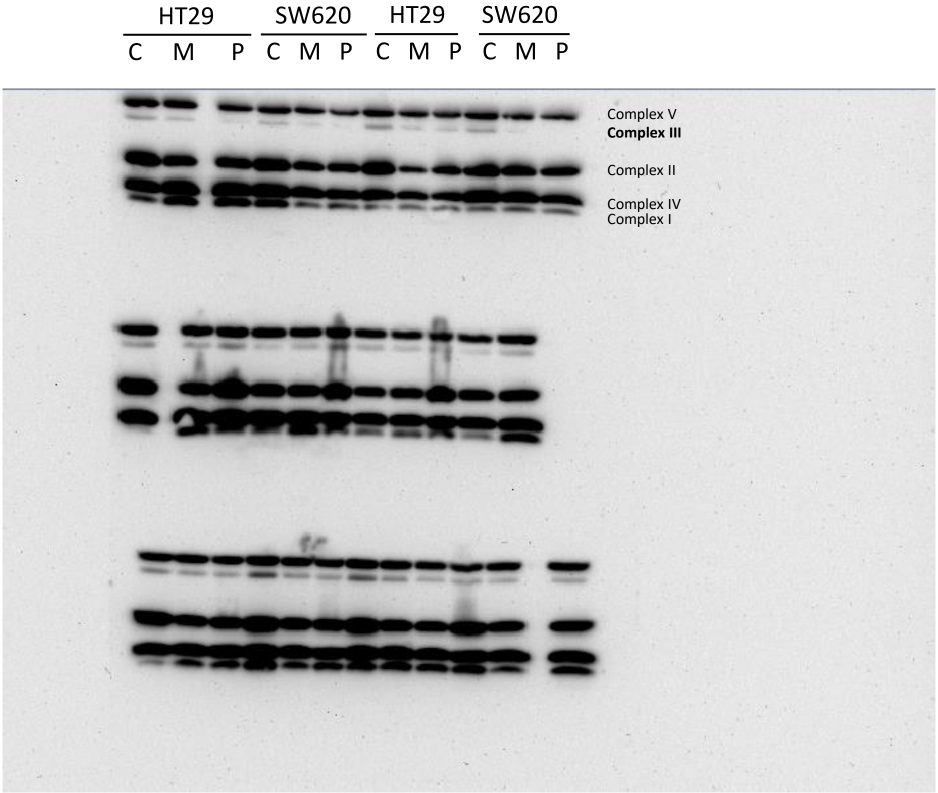

C: vehicle-treated cells
M: miltefosine-treated cells
P: perifosine-treated cells

3 gels blotted into the same membrane. All gels follow the same order.

Blot - GAPDH complexes (Figure 3)

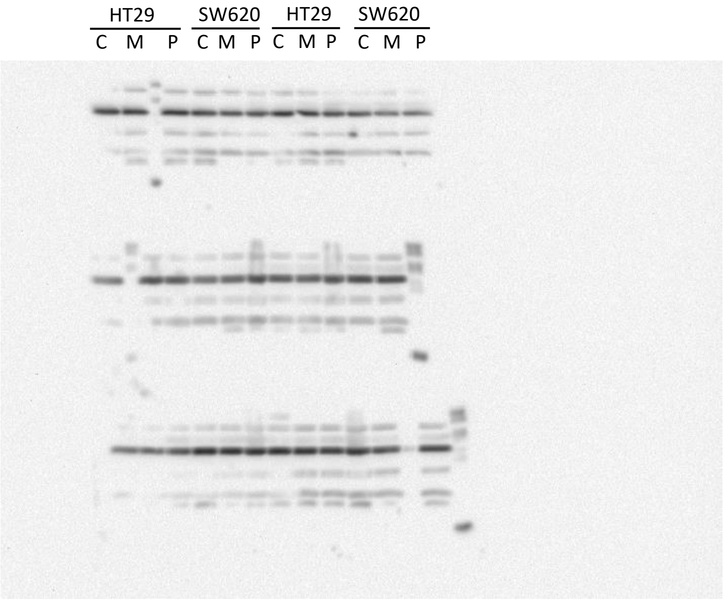

C: vehicle-treated cells
M: miltefosine-treated cells
P: perifosine-treated cells

3 gels blotted into the same membrane. All gels follow the same order.
NOTE: This blot was performed after stripping of OXPHOS membrane.

Blot – PDH (Figure 4)

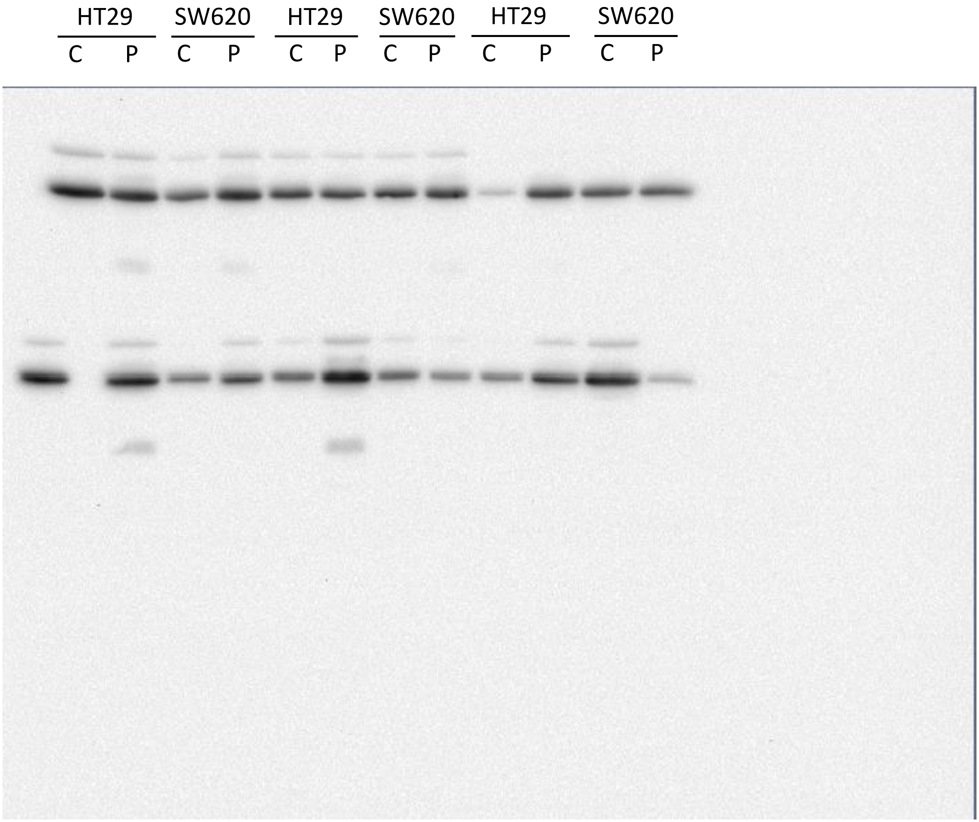

C: vehicle-treated cells
P: perifosine-treated cells

2 gels blotted into the same membrane. All gels follow the same order.

Blot – IDH (Figure 4)

HT29 SW620 HT29 SW620 HT29 SW620

C P C P C P C P C P C P

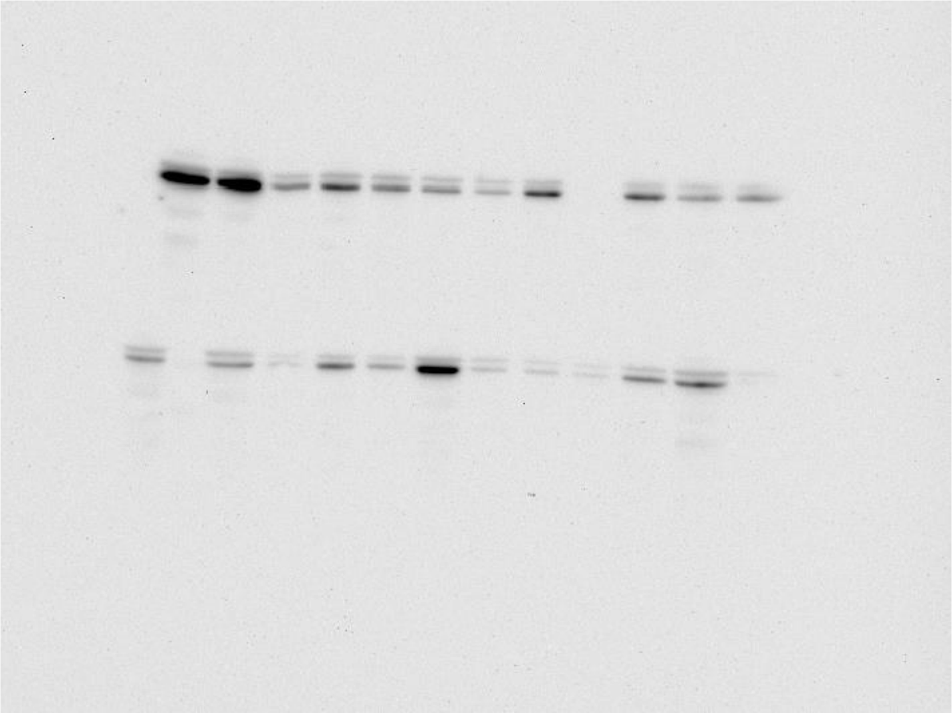

C: vehicle-treated cells
P: perifosine-treated cells

2 gels blotted into the same membrane. All gels follow the same order.

Blot – UCP2 (Figure 4)

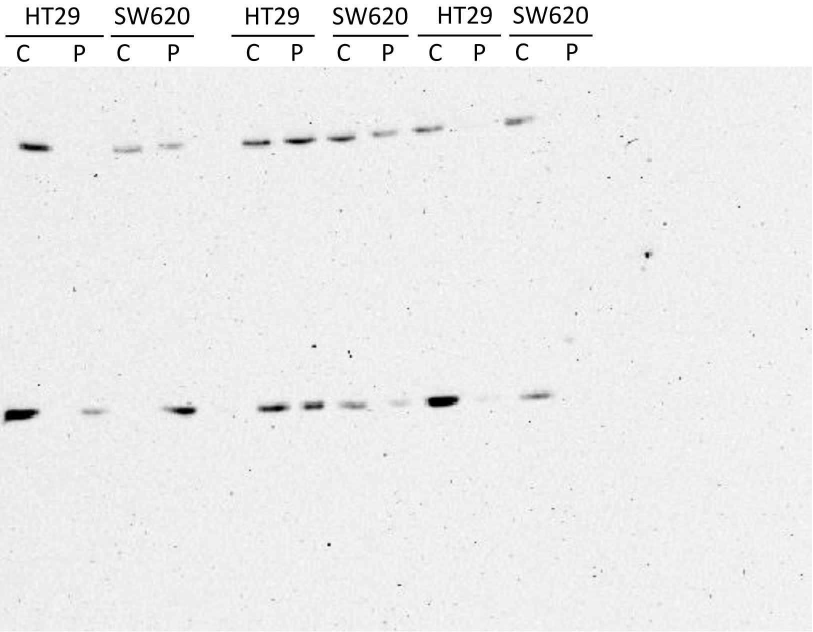

C: vehicle-treated cells
P: perifosine-treated cells

2 gels blotted into the same membrane. All gels follow the same order.

Blot – GAPDH (Figures 4 and 6)

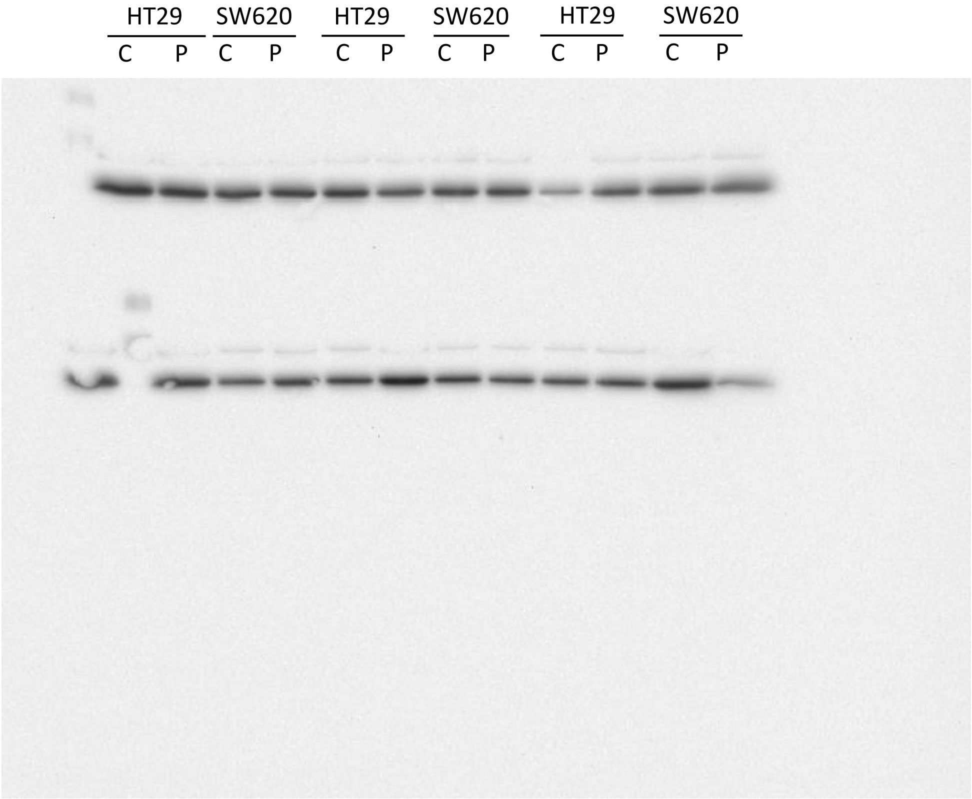

C: vehicle-treated cells
P: perifosine-treated cells

2 gels blotted into the same membrane. All gels follow the same order.

Blot – PARP (Figure 6)

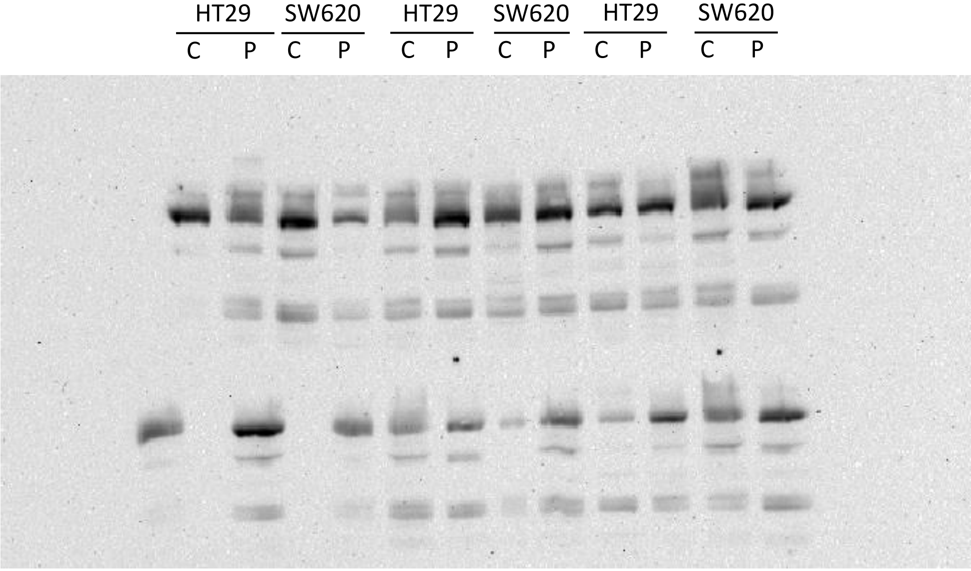

C: vehicle-treated cells
P: perifosine-treated cells

2 gels blotted into the same membrane. All gels follow the same order.

Blot – LC3 (Figure 6)

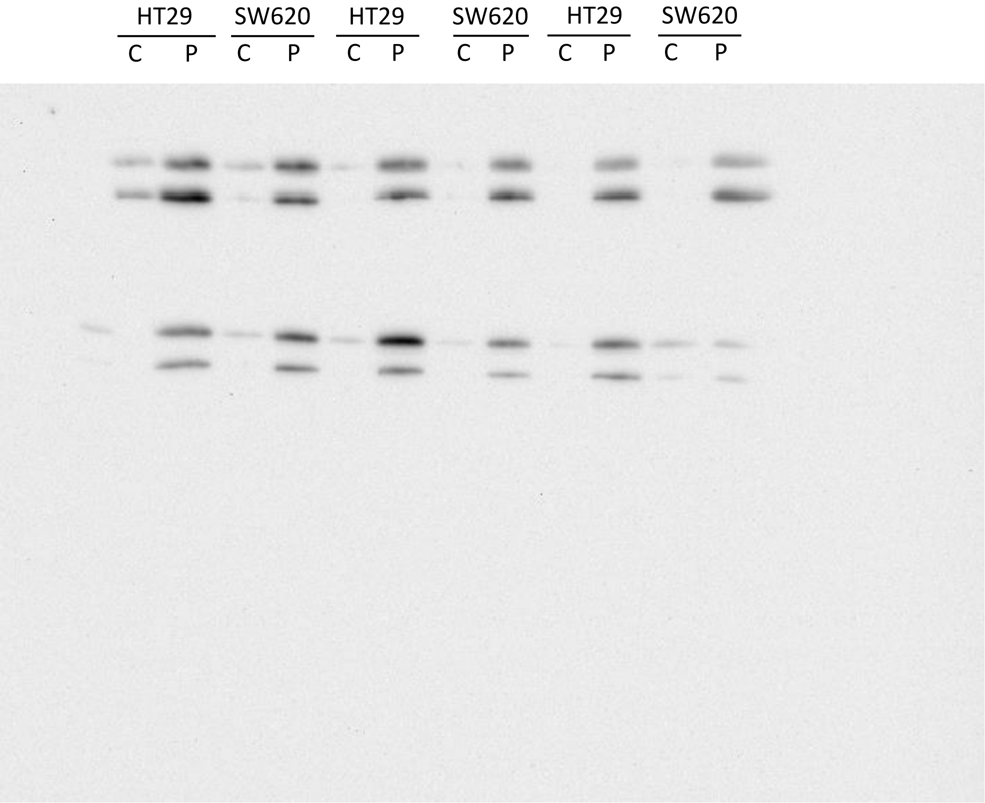

C: vehicle-treated cells
P: perifosine-treated cells

2 gels blotted into the same membrane. All gels follow the same order.
